# Supplementary material for: Modeling glioblastoma heterogeneity as a dynamic network of cell states
Source: Mol Syst Biol. 2021 Sep 16;17(9):e10105. doi: 10.15252/msb.202010105 (PMC8444284; doi:10.15252/msb.202010105)
Supplement: Supplementary file 5 — Source Data for Figure 3 [file MSB-17-e10105-s001.zip › Figure3A_sourcedata/GSEA_3065/nosigdb_state5.GseaPreranked.1622718435914/DOROSZKO_U3054MG_CLUSTER_5.html]

Details for gene set DOROSZKO\_U3054MG\_CLUSTER\_5[GSEA]

|  || Dataset | state5 |
| Phenotype | NoPhenotypeAvailable |
| Upregulated in class | na\_pos |
| GeneSet | DOROSZKO\_U3054MG\_CLUSTER\_5 |
| Enrichment Score (ES) | 0.49212506 |
| Normalized Enrichment Score (NES) | 1.9534379 |
| Nominal p-value | 0.0 |
| FDR q-value | 0.0020026546 |
| FWER p-Value | 0.013 |
Table: GSEA Results Summary

  

Fig 1: Enrichment plot: DOROSZKO\_U3054MG\_CLUSTER\_5      
 Profile of the Running ES Score & Positions of GeneSet Members on the Rank Ordered List

  

| PROBE | GENE SYMBOL | GENE\_TITLE | RANK IN GENE LIST | RANK METRIC SCORE | RUNNING ES | CORE ENRICHMENT || 1 | SPPL2B |  |  | 66 | 0.587 | 0.0329 | Yes |
| 2 | DDR1 |  |  | 81 | 0.551 | 0.0691 | Yes |
| 3 | LRP6 |  |  | 105 | 0.521 | 0.1022 | Yes |
| 4 | ITGB8 |  |  | 126 | 0.503 | 0.1345 | Yes |
| 5 | LINC00685 |  |  | 158 | 0.476 | 0.1637 | Yes |
| 6 | SCD5 |  |  | 173 | 0.460 | 0.1937 | Yes |
| 7 | SYT11 |  |  | 265 | 0.400 | 0.2109 | Yes |
| 8 | DAG1 |  |  | 373 | 0.351 | 0.2230 | Yes |
| 9 | FREM2 |  |  | 377 | 0.349 | 0.2466 | Yes |
| 10 | RETREG1 |  |  | 403 | 0.340 | 0.2671 | Yes |
| 11 | SERINC5 |  |  | 477 | 0.318 | 0.2807 | Yes |
| 12 | SLC22A17 |  |  | 550 | 0.297 | 0.2930 | Yes |
| 13 | NTRK2 |  |  | 587 | 0.288 | 0.3087 | Yes |
| 14 | SPECC1 |  |  | 689 | 0.266 | 0.3156 | Yes |
| 15 | HTRA1 |  |  | 690 | 0.266 | 0.3339 | Yes |
| 16 | PDPN |  |  | 719 | 0.258 | 0.3484 | Yes |
| 17 | NRCAM |  |  | 748 | 0.251 | 0.3625 | Yes |
| 18 | CELSR2 |  |  | 750 | 0.251 | 0.3795 | Yes |
| 19 | DPY19L1 |  |  | 775 | 0.246 | 0.3937 | Yes |
| 20 | PTPRE |  |  | 893 | 0.227 | 0.3962 | Yes |
| 21 | ILDR2 |  |  | 965 | 0.215 | 0.4029 | Yes |
| 22 | C1orf61 |  |  | 1057 | 0.204 | 0.4067 | Yes |
| 23 | PLPP2 |  |  | 1111 | 0.197 | 0.4142 | Yes |
| 24 | SEMA3E |  |  | 1196 | 0.185 | 0.4175 | Yes |
| 25 | CRB2 |  |  | 1230 | 0.181 | 0.4262 | Yes |
| 26 | COL16A1 |  |  | 1291 | 0.173 | 0.4313 | Yes |
| 27 | DNM3 |  |  | 1294 | 0.172 | 0.4429 | Yes |
| 28 | FLRT3 |  |  | 1417 | 0.160 | 0.4401 | Yes |
| 29 | CNKSR3 |  |  | 1468 | 0.155 | 0.4452 | Yes |
| 30 | ENDOD1 |  |  | 1552 | 0.147 | 0.4459 | Yes |
| 31 | SEMA3B |  |  | 1604 | 0.141 | 0.4498 | Yes |
| 32 | LRRTM2 |  |  | 1627 | 0.140 | 0.4569 | Yes |
| 33 | EPN2 |  |  | 1688 | 0.134 | 0.4594 | Yes |
| 34 | ADGRE5 |  |  | 1730 | 0.131 | 0.4638 | Yes |
| 35 | PIEZO1 |  |  | 1786 | 0.128 | 0.4664 | Yes |
| 36 | NCKAP5 |  |  | 1790 | 0.127 | 0.4748 | Yes |
| 37 | CERCAM |  |  | 1837 | 0.124 | 0.4781 | Yes |
| 38 | KCND3 |  |  | 1838 | 0.123 | 0.4865 | Yes |
| 39 | NLGN3 |  |  | 1947 | 0.115 | 0.4823 | Yes |
| 40 | ABCA2 |  |  | 1975 | 0.113 | 0.4870 | Yes |
| 41 | NFASC |  |  | 1999 | 0.112 | 0.4921 | Yes |
| 42 | ABCA1 |  |  | 2142 | 0.103 | 0.4832 | No |
| 43 | PLEKHA1 |  |  | 2330 | 0.090 | 0.4683 | No |
| 44 | SLCO3A1 |  |  | 2370 | 0.088 | 0.4700 | No |
| 45 | ELOVL1 |  |  | 2461 | 0.082 | 0.4655 | No |
| 46 | SHISA9 |  |  | 2493 | 0.080 | 0.4674 | No |
| 47 | TLE4 |  |  | 2935 | 0.056 | 0.4217 | No |
| 48 | SLC22A23 |  |  | 3276 | 0.038 | 0.3860 | No |
| 49 | ZNF516 |  |  | 3341 | 0.034 | 0.3812 | No |
| 50 | FRMD5 |  |  | 3383 | 0.032 | 0.3788 | No |
| 51 | APLP1 |  |  | 3430 | 0.030 | 0.3757 | No |
| 52 | ARC |  |  | 3455 | 0.029 | 0.3750 | No |
| 53 | CXCL16 |  |  | 3689 | 0.020 | 0.3502 | No |
| 54 | KIF1B |  |  | 3759 | 0.017 | 0.3436 | No |
| 55 | DIP2B |  |  | 3861 | 0.013 | 0.3331 | No |
| 56 | MAMLD1 |  |  | 3895 | 0.012 | 0.3302 | No |
| 57 | SORBS1 |  |  | 4146 | 0.001 | 0.3022 | No |
| 58 | SOX8 |  |  | 4306 | -0.006 | 0.2847 | No |
| 59 | MED15 |  |  | 4408 | -0.010 | 0.2741 | No |
| 60 | IPO13 |  |  | 4468 | -0.013 | 0.2683 | No |
| 61 | PIM3 |  |  | 4517 | -0.015 | 0.2639 | No |
| 62 | DUSP16 |  |  | 4557 | -0.016 | 0.2606 | No |
| 63 | PLEKHA4 |  |  | 4570 | -0.017 | 0.2605 | No |
| 64 | DIRAS1 |  |  | 4701 | -0.022 | 0.2473 | No |
| 65 | ASPHD1 |  |  | 4793 | -0.025 | 0.2388 | No |
| 66 | EN2 |  |  | 4832 | -0.027 | 0.2364 | No |
| 67 | TST |  |  | 4856 | -0.028 | 0.2357 | No |
| 68 | ANKS1B |  |  | 5003 | -0.033 | 0.2216 | No |
| 69 | GADD45B |  |  | 5106 | -0.037 | 0.2126 | No |
| 70 | RALGDS |  |  | 5182 | -0.039 | 0.2069 | No |
| 71 | FBXO21 |  |  | 5379 | -0.048 | 0.1881 | No |
| 72 | FAM107B |  |  | 5545 | -0.055 | 0.1733 | No |
| 73 | KCNMB4 |  |  | 5714 | -0.062 | 0.1587 | No |
| 74 | ISG20 |  |  | 5757 | -0.064 | 0.1584 | No |
| 75 | C12orf76 |  |  | 5798 | -0.065 | 0.1584 | No |
| 76 | FAM19A5 |  |  | 5803 | -0.066 | 0.1624 | No |
| 77 | DHX40 |  |  | 5864 | -0.068 | 0.1603 | No |
| 78 | PPP2R2A |  |  | 5883 | -0.069 | 0.1630 | No |
| 79 | SHISA4 |  |  | 5957 | -0.072 | 0.1598 | No |
| 80 | GATM |  |  | 6035 | -0.076 | 0.1563 | No |
| 81 | C1orf198 |  |  | 6097 | -0.079 | 0.1549 | No |
| 82 | MID1IP1 |  |  | 6270 | -0.089 | 0.1417 | No |
| 83 | OLIG2 |  |  | 6595 | -0.108 | 0.1126 | No |
| 84 | CNIH3 |  |  | 6644 | -0.110 | 0.1148 | No |
| 85 | SERTAD2 |  |  | 6719 | -0.115 | 0.1144 | No |
| 86 | TIPARP |  |  | 6731 | -0.116 | 0.1211 | No |
| 87 | CDC42EP1 |  |  | 6781 | -0.119 | 0.1237 | No |
| 88 | CDK16 |  |  | 7148 | -0.150 | 0.0929 | No |
| 89 | SOX21 |  |  | 7198 | -0.155 | 0.0980 | No |
| 90 | PLEKHB1 |  |  | 7506 | -0.191 | 0.0766 | No |
| 91 | HPCAL1 |  |  | 7544 | -0.196 | 0.0859 | No |
| 92 | GLUL |  |  | 7594 | -0.203 | 0.0944 | No |
| 93 | CARD19 |  |  | 7820 | -0.238 | 0.0854 | No |
| 94 | RND2 |  |  | 7823 | -0.239 | 0.1016 | No |
| 95 | LYPD1 |  |  | 8476 | -0.422 | 0.0571 | No |
Table: GSEA details [plain text format]

  

Fig 2: DOROSZKO\_U3054MG\_CLUSTER\_5: Random ES distribution      
 Gene set null distribution of ES for **DOROSZKO\_U3054MG\_CLUSTER\_5**
